# Autonomous radiotherapy planning via agentic orchestration using a multimodal TPS-integrated compound AI platform

**DOI:** 10.1088/3049-477X/ae7978

**Published:** 2026-07-15

**Authors:** Austen Matthew Maniscalco, Yang Kyun Park, Sean J Domal, Mu-Han Lin, Sanda Harabagiu, Steve B Jiang, Dan Nguyen

**Affiliations:** 1 Medical Artificial Intelligence and Automation (MAIA) Lab, UT Southwestern Medical Center, Dallas, TX, United States of America; 2 Department of Radiation Oncology, UT Southwestern Medical Center, Dallas, TX, United States of America; 3 University of Texas at Dallas, Richardson, TX, United States of America

**Keywords:** autonomous treatment planning, radiation therapy, dose prediction, compound AI systems, LLM agents, multi-agent orchestration, agentic RAG

## Abstract

*Purpose.* Radiation therapy (RT) treatment planning requires iterative, multi-day optimization workflows in which subjective planning strategies produce inter-planner variability in plan quality. Existing computational approaches automate isolated aspects of this workflow, yet none orchestrates an end-to-end pipeline from physician directive to deliverable plan. We developed a compound artificial intelligence (AI) platform for fully autonomous RT treatment planning that combines multi-agent large language model (LLM) orchestration with directive-conditioned three-dimensional (3D) dose prediction, natively integrated with a commercial treatment planning system (TPS). *Methods.* Seven specialized agents navigated the multi-objective optimization landscape through structured clinical reasoning, iteratively analyzing dose-volume histogram (DVH) metrics and spatial dose patterns, formulating trade-off strategies, and executing validated modifications through the TPS across five fully autonomous iterations per case. A directive-conditioned 3D dose prediction model supplied patient-specific DVH values from which initial optimization objectives were autonomously derived, eliminating the need for curated templates or manual initialization. A retrieval-augmented generation (RAG) system encoded institutional knowledge into the planning workflow. We evaluated 60 retrospective cases across brain, lung, and prostate sites, with 10 intensity-modulated RT (IMRT) and 10 volumetric modulated arc therapy (VMAT) plans per site spanning 20.0–79.2 Gy in 3–44 fractions, scored by the proportion of dosimetric criteria satisfied. *Results.* Across all 60 cases, AI plans achieved 89.8$\pm$9.4% of dosimetric criteria versus 85.2$\pm$10.8% for clinical reference plans ($p < 0.001$). IMRT plans improved in 25 of 30 cases with none worsened (94.1$\pm$6.7% vs 84.3$\pm$11.8%, $p < 0.001$); VMAT plans showed no significant difference (85.6$\pm$9.9% vs 86.1$\pm$9.7%, $p = 0.770$). Each plan iteration completed in $20.2\pm 12.7$ min, of which agent reasoning consumed $5.2\pm 1.7$ min ($114{,}429\pm 11{,}798$ tokens, $0.43 \pm 0.04$). *Conclusions.* These results established the feasibility of end-to-end, fully autonomous, universal RT treatment planning through compound AI. Integrating dose prediction as an agent-invoked tool for objective initialization resolved the dependency on curated templates and manual specification that constrained prior LLM-based planning systems.

## 1. Introduction

### 1.1. Clinical context and the manual planning bottleneck

Radiation therapy (RT) is a cornerstone of modern cancer care, prescribed to over 50% of all cancer patients worldwide [1, 2]. The quality of each treatment plan directly influences clinical outcomes including tumor control probability, normal tissue complication rates, and quality of life [3–5]. Treatment planning designs a patient-specific radiation delivery strategy by tailoring hundreds of thousands of parameters that control beam geometry, aperture shape, energy, and intensity to each patient’s anatomy and clinical dose constraints [6]. Each organ at risk (OAR) has specific radiation tolerances that may conflict with target coverage objectives, creating an inherently multi-objective optimization problem in which dose reduction to one structure may compromise another [7–9]. Poorly resolved trade-offs drive treatment-related toxicity and diminished quality of life [10, 11]. Ideally, each plan would be Pareto-optimal (no objective improvable without degrading another), yet consistently achieving this in clinical practice remains a persistent challenge.

RT planning is a sequential, multidisciplinary workflow whose inputs are established during computed tomography (CT) simulation, in which a CT scan is acquired under a reproducible treatment position to capture patient anatomy and supply the tissue properties required for dose calculation [12]. A radiation oncologist delineates planning target volumes (PTVs) and surrounding OARs on this image and issues a dosimetric directive specifying the prescription dose and normal tissue dose limits. A treatment planner then drives the inverse optimization engine of a treatment planning system (TPS), iteratively adjusting beam parameters and objective weights until a clinically acceptable plan emerges [13]. Each optimization cycle typically requires 5–30 min of computation, and repeated iterations are needed as the planner navigates trade-offs [14]. Iterative physician-planner refinement typically extends the total timeline to 3–7 d, longer for complex cases [15–18]. The dependence on subjective judgment and multidisciplinary coordination makes RT planning a critical bottleneck in cancer care.

### 1.2. Variability, efficiency, and scalability pressures on planning

Plan quality varies substantially across planners, commonly attributed to differences in experience, available time, and physician-planner communication [19]. However, empirical evidence complicates this assumption. Inter-planner variability persists across both inter- and intra-institutional settings [20]. An inter-institutional study of 125 plans generated worldwide for a single prostate patient found wide quality variability with no meaningful correlation to TPS, technique, planner education, certification, years of experience, or self-reported confidence [21]. An intra-institutional study reinforced this: 40 planners across five campuses produced plans scoring from 80.24 to 135.89 [22]. These findings suggest variability stems from subjective optimization strategy rather than measurable qualifications. Beyond this variability in plan quality, the time required to produce plans carries its own clinical cost. Delays between diagnosis and the initiation of RT are associated with reduced tumor control probability and decreased survival across multiple disease sites [23, 24]. Treatment delays reduce local control by approximately 8% per month in head and neck cancer, rising to approximately 20% per month for the most rapidly proliferating tumors [25]. A separate head and neck cohort observed median tumor volume growth of 3.2% per day and a doubling time of 19 d [26]. Patient anatomy can therefore change substantially during extended planning timelines, rendering carefully optimized plans suboptimal by the time treatment begins.

These pressures will only intensify as RT demand scales. The manual planning workflow faces fundamental scalability limits as RT adoption grows globally and treatment complexity rises with advanced techniques such as stereotactic treatments and adaptive radiotherapy (ART) [27–30]. Global RT demand is projected to outpace workforce capacity, requiring over 60% expansion by 2050 [31]. Resource-limited institutions are already affected, often lacking enough experienced planners to handle complex cases efficiently [32]. ART compounds these pressures further, as the re-planning demands driven by anatomical changes during treatment could overwhelm existing capacity [33]. Automation of routine planning tasks is a route to efficient and consistent plan quality at scale, sustaining global access to high-quality RT.

### 1.3. Existing computational approaches and their limitations

Multiple computational approaches have attempted to automate aspects of treatment planning, yet each suffers from fundamental constraints that prevent comprehensive end-to-end deployment. Knowledge-based planning (KBP) leverages historical plan data to estimate achievable dose-volume histogram (DVH) curves for new patients by matching their anatomy to previously treated cases [34, 35]. From a curated cohort of prior plans, the approach extracts handcrafted geometric and dosimetric features and fits regression models that predict cumulative DVH curves for organs at risk [36, 37]. However, KBP is constrained at the input, output, and optimization stages. Its handcrafted input features collapse complex three-dimensional anatomy into pairwise summaries between individual organs and the target. Its DVH outputs lack the spatial context that volumetric methods preserve natively. Even when these outputs are accurate, the inverse optimizer may converge to inferior local minima, forcing the planner back into manual parameter adjustment [38]. KBP therefore narrows the optimization search space but does not automate the iterative manual workflow that drives planning delays.

Multi-criteria optimization (MCO) enables navigation of the trade-off space by generating alternative plans along an approximate Pareto surface for specified regions of interest [39]. Once a fully optimized base plan anchors the surface, planners can rapidly visualize alternative trade-off strategies without repeated full optimizations. However, MCO is constrained at every stage of this workflow. Producing the base plan still demands considerable upfront manual effort, and exploration of the resulting Pareto surface can be slow and computationally expensive [40]. In addition, selection of a final plan cannot be trivially automated because Pareto-optimal plans are not guaranteed to be clinically ideal [41]. MCO therefore restructures how planners navigate trade-offs but does not reduce the manual effort required to produce and select a clinically acceptable plan.

Deep learning (DL) models have been developed to estimate clinically realistic 3D dose distributions directly from patient anatomy and treatment parameters without requiring full inverse optimization [42–44]. Convolutional neural networks trained on prior plans learn the mapping from volumetric anatomical input to feasible dose output, and the resulting predictions can guide and accelerate the downstream planning process. One information-aided scheme seeded optimization objectives with predicted dose values and reduced manual planning time by nearly 50% [45]. Predicted distributions have also served as automated quality assurance references for head and neck plans [46]. However, dose prediction is constrained at both ends of the planning pipeline. A predicted distribution is not itself a deliverable plan and must still be translated into machine parameters through inverse optimization. Typical predictors are also trained and validated on a single disease site and prescription, requiring a new model for each site-prescription combination and creating a scaling and maintenance burden that limits clinical practicality. DL-based dose prediction therefore provides a powerful auxiliary signal but does not close the loop from patient anatomy to clinically deliverable plan.

Reinforcement learning (RL) approaches have been investigated for automating the adjustment of optimization parameters through trial-and-error learning, mimicking the decision-making process of expert planners [47–49]. A neural network is trained to observe current plan metrics and select parameter modifications that maximize a reward signal based on changes in dosimetric quality. However, RL is constrained at every component of this learning loop [50]. The state representation typically relies on DVH metrics and scalar dose statistics, which cannot capture spatially dependent features that influence clinical decision-making. The reward function is inherently difficult to specify, as it must serve as a quantitative surrogate for a physician’s multi-factorial judgment of plan quality. The action space is also challenging to scale as the number of possible modifications grows. A comprehensive review further identified training inefficiency, limited methods for quality assessment, and poor interpretability of learned policies as persistent barriers [51]. RL therefore offers a plausible automation mechanism but is difficult to generalize across diverse clinical cases and to reconcile with the interpretability standards required in a safety-critical domain.

### 1.4. LLM agents for radiotherapy treatment planning

Recent advances in LLMs have enabled a new class of autonomous software agents that orchestrate complex technical workflows by interpreting goals, planning multi-step actions, and refining outputs through self-directed reasoning [52–55]. Applied to RT treatment planning, LLM agents equipped with TPS scripting access can transform the manual trial-and-error workflow into an autonomous optimization process with interpretable reasoning traces that enable clinical oversight without continuous human supervision. Such an agent can parse physician directives to extract both explicit constraints (e.g. ‘parotid mean dose $ < $26 Gy’) and implicit preferences (e.g. ‘prioritize xerostomia reduction’). It can then systematically explore the trade-off landscape through TPS tool calls and iteratively adjust optimization parameters based on DVH results. The resulting natural-language reasoning trace contrasts directly with the opaque policies learned by RL and enables clinicians to audit each decision as it is made.

Several recent investigations have demonstrated the feasibility of LLM-based treatment planning automation. Wang *et al* proposed GPT-Plan, a multi-agent pipeline mimicking the collaborative workflow of a dosimetrist and physicist, and evaluated it on 12 lung and 5 cervical cases with conventionally fractionated prescriptions [56]. Liu *et al* introduced GPT-RadPlan, a multimodal approach using a vision-capable LLM to evaluate DVH data and dose distribution images, and evaluated it on 17 head and neck and 13 prostate cases with conventionally fractionated prescriptions [57]. Nusrat *et al* developed DOLA, demonstrating that a locally hosted LLM (LLaMa 3.1) could perform prostate plan optimization on 18 patients all prescribed 60 Gy in 20 fractions [58]. Yang *et al* employed a zero-shot approach in which an LLM agent with no prior exposure to manually generated plans iteratively adjusted inverse optimization constraints via direct TPS API on 20 head and neck cases all prescribed 70 Gy in 35 fractions [59]. Wei *et al* performed a comparative evaluation of multiple LLMs for cervical cancer planning on 35 patients all receiving 36 Gy in 20 fractions [60]. Nusrat *et al* subsequently introduced SAGE, a human-in-the-loop reasoning agent for stereotactic radiosurgery planning, evaluated on 41 brain metastasis patients receiving 18 Gy in 1 fraction [61].

Collectively, these studies establish that LLM-based reasoning can navigate the multi-objective optimization landscape with interpretable decision-making. However, several limitations persist across this body of work. All existing approaches were restricted to 1 or 2 disease sites and evaluated on homogeneous cohorts with a single prescription and fractionation scheme per study. Performance across heterogeneous prescriptions remains untested, precluding disease-site-agnostic generalization. Multi-agent coordination and structured mechanisms for real-time end-user oversight during autonomous planning also remain underdeveloped.

Most critically, none of these methods offers an autonomous mechanism for producing an informed starting point for optimization. Without an informed starting point, the inverse solver is prone to wandering through inferior regions of the search space and failing to converge toward the Pareto-optimal frontier. Downstream agent reasoning cannot reliably correct this trajectory. The agent has no patient-specific signal of what is achievable and must instead rely on prior context that may not align with the case at hand.

Current workarounds do not resolve this gap. Handcrafted static templates for objective initialization struggle to generalize, as each patient’s target volume differs in physical location, size, and overlap with surrounding organs. A separate template would also be required for every site-prescription combination. Human-supplied starting points reintroduce manual expert effort at the very step where an autonomous agent’s value should be most apparent. Existing methods therefore face a forced choice between uninformed initialization that compromises plan quality and pseudo-informed initialization that sacrifices autonomy or scalability.

### 1.5. Agentic AI and compound AI systems

More broadly, the concept of LLM-based autonomous agents extends beyond single-model inference to encompass systems that plan, decide, recall, reflect, and leverage specialized tools in pursuit of complex goals [53, 62]. This represents an architectural shift from passive prediction models to active problem-solving agents that exhibit goal-directed behavior. Such agentic artificial intelligence (AI) systems hold particular potential in medicine and healthcare, where they can automate clinical workflows, support multi-step clinical reasoning, and collaborate across specialized agent roles [63].

The compound AI paradigm represents a further evolution. In this paradigm, LLM orchestration layers coordinate multiple specialized models and tools to achieve capabilities that exceed what either component achieves independently [64]. The LLM agent provides high-level reasoning, planning, and natural language interaction. It invokes specialized DL models for computationally intensive predictions and scripting tools for system-level operations. This architectural approach is particularly well-suited to RT treatment planning, where reasoning, prediction, and optimization must be coordinated across diverse anatomical contexts.

Despite this potential, existing computational approaches address only isolated tasks. No current system autonomously orchestrates a complete planning workflow from directive interpretation through deliverable plan generation.

Closing this gap requires progress along three axes. First, an autonomous agent requires a robust and generalizable dose predictor that can provide spatially informed initial objectives across disease sites and prescriptions. Second, the system must be capable of reading and processing patient-specific clinical data and interfacing directly with commercial TPS infrastructure. Third, multi-agent coordination must integrate these components within a unified platform that supports clinical reasoning, iterative optimization, and expert oversight. The unification of these capabilities would enable the transition from AI-assisted to AI-autonomous planning.

### 1.6. Universal dose prediction

As previously established, typical DL dose predictors face two limitations: a predicted distribution is not itself a deliverable plan, and each model is tied to a single site-prescription combination. A compound AI architecture can resolve the first limitation by treating the predictor as an agent-invoked tool whose output seeds or warm-starts downstream inverse optimization. The second limitation is critical to address in an agent-driven platform. If the dose predictor only works on the sites and prescriptions it was trained on, the platform inherits the same per-combination retraining burden that prevents standalone dose predictors from scaling clinically. An agent-invoked tool must instead generalize to whatever case the agent is handed.

To address this constraint, we previously developed the framework for a universal dose prediction model that generalizes across disease sites and prescriptions [65, 66]. Prior anatomy-only approaches required the model to implicitly learn dosimetric patterns from the training data, which limited generalization across disease sites and fractionation schemes [42–44]. Our directive-conditioned architecture instead encodes dosimetric goals as explicit input channels, enabling disease-site-agnostic and prescription-agnostic inference from a single trained model.

Because the predictor serves as a warm-start tool rather than the final answer, it need only provide a reasonable initial objective configuration from which the agentic optimization loop iteratively refines toward clinical acceptability. This capability enables a single dose predictor to be invoked as a tool by the orchestration layer regardless of disease site or fractionation. It eliminates the per-site model-management burden that would otherwise preclude clinical deployment of a general-purpose autonomous planning platform.

### 1.7. Purpose

Building on this foundation, we developed a compound AI platform that coordinates multi-agent LLM reasoning with a site- and prescription-agnostic 3D dose predictor for autonomous RT treatment planning. The platform is natively integrated with a commercial TPS through its scripting API. It aims to address the planning bottleneck through autonomous assistance and reduce inter-planner variability through plan quality standardization. It also aims to mitigate the scalability limitations of the manual workflow. To our knowledge, this is the first system to integrate multi-agent clinical reasoning with DL-based dose prediction as an agent-invoked tool. It also supports autonomous objective initialization from institutional knowledge and real-time expert steering within a single clinical platform. We evaluated the platform on 60 retrospective cases spanning brain, lung, and prostate sites. Each site included 10 intensity-modulated RT (IMRT) and 10 volumetric modulated arc therapy (VMAT) cases with no restriction on prescription dose or fractionation. Autonomous plan quality was assessed relative to clinical reference plans.

## 2. Methods

### 2.1. System overview

**Figure 1.**
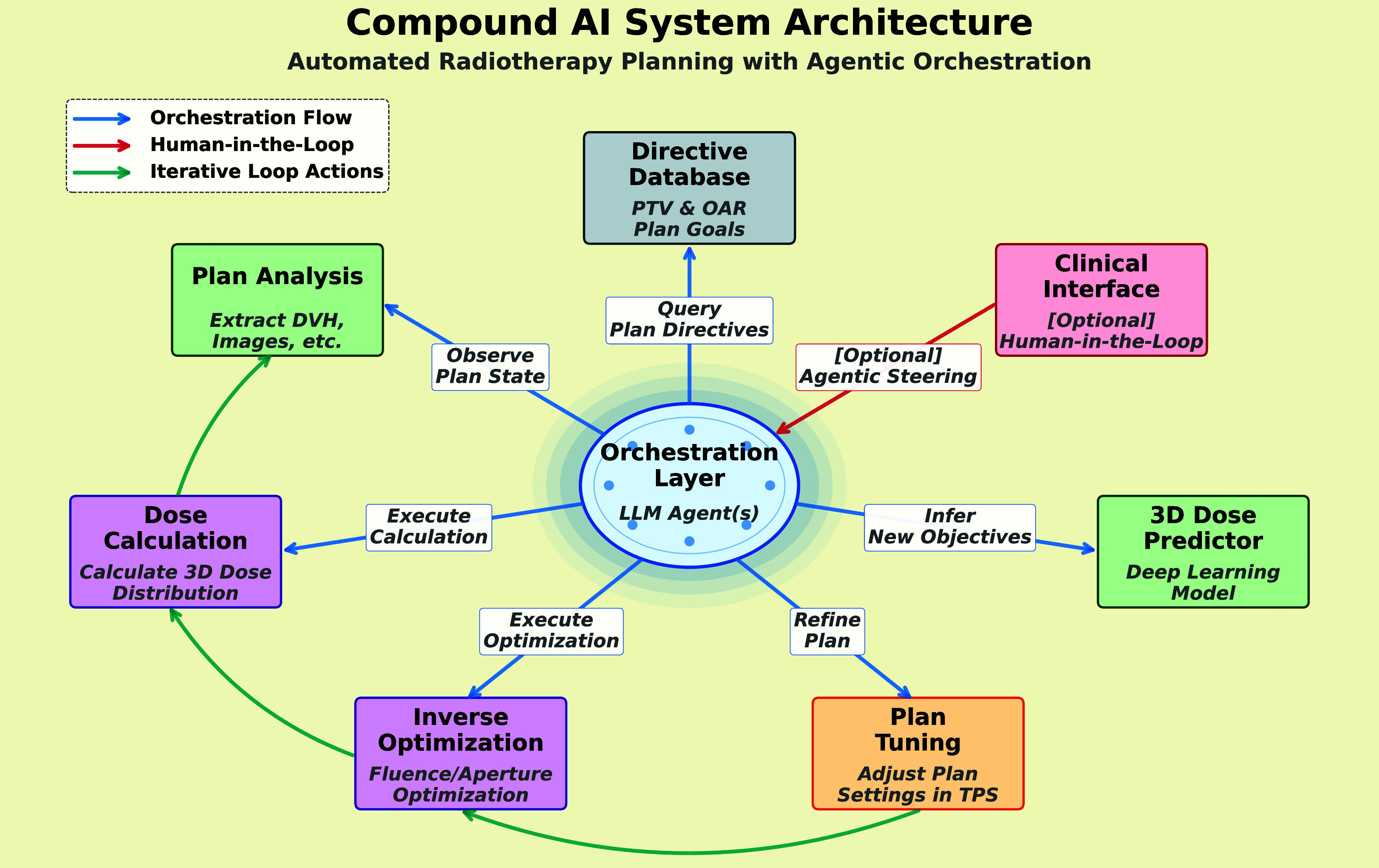
Compound artificial intelligence (AI) platform architecture. A central agentic orchestration layer coordinates specialized modules via direct tool invocation. Blue arrows indicate orchestration-initiated operations (directive queries, dose prediction, plan modification, optimization/calculation, and plan data analysis extraction). The red arrow represents optional expert steering through the human-in-the-loop interface. Green arrows indicate iterative loop actions. The agentic orchestration layer selects tools based on planning context to iteratively refine plans toward clinical acceptability, interfacing with the treatment planning system (TPS) for optimization and dose-volume histogram (DVH) extraction.

The compound AI platform architecture is site-agnostic and operates end-to-end from directive interpretation through generation of a deliverable plan. This layered design preserves end-user oversight at each stage while enabling fully autonomous operation, as illustrated in figure 1.

**Figure 2.**
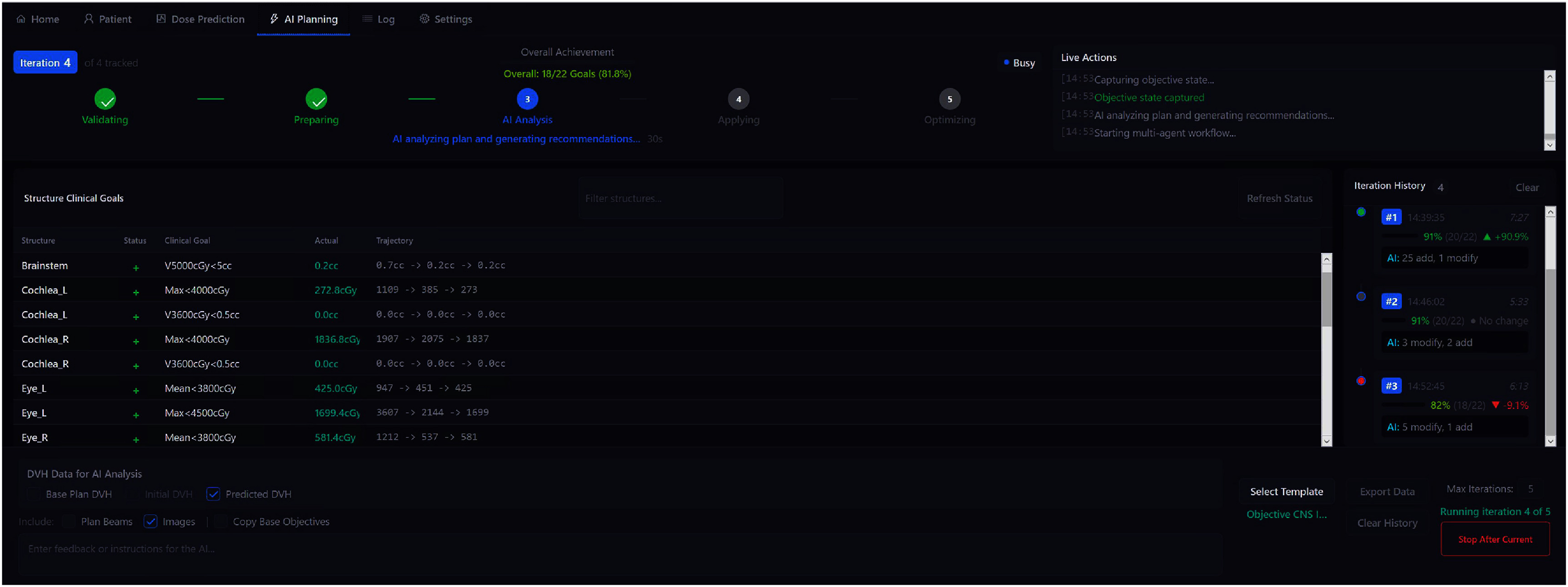
The platform interface during an autonomous planning session. The left panel displays goal achievement status and per-structure constraint trajectories, while the right panel shows live agent actions and iteration history. Real-time monitoring enables end-user oversight of the autonomous optimization process.

The platform comprises four core components: a multi-agent orchestration pipeline for clinical reasoning, a directive-conditioned 3D dose prediction model for objective initialization, an isolated host process exposing TPS operations through its scripting API, and a retrieval-augmented generation (RAG) system for institutional knowledge retrieval. A graphical user interface enables end-user interaction and real-time monitoring throughout the planning session (figure 2).

### 2.2. Multi-agent LLM architecture

**Figure 3.**
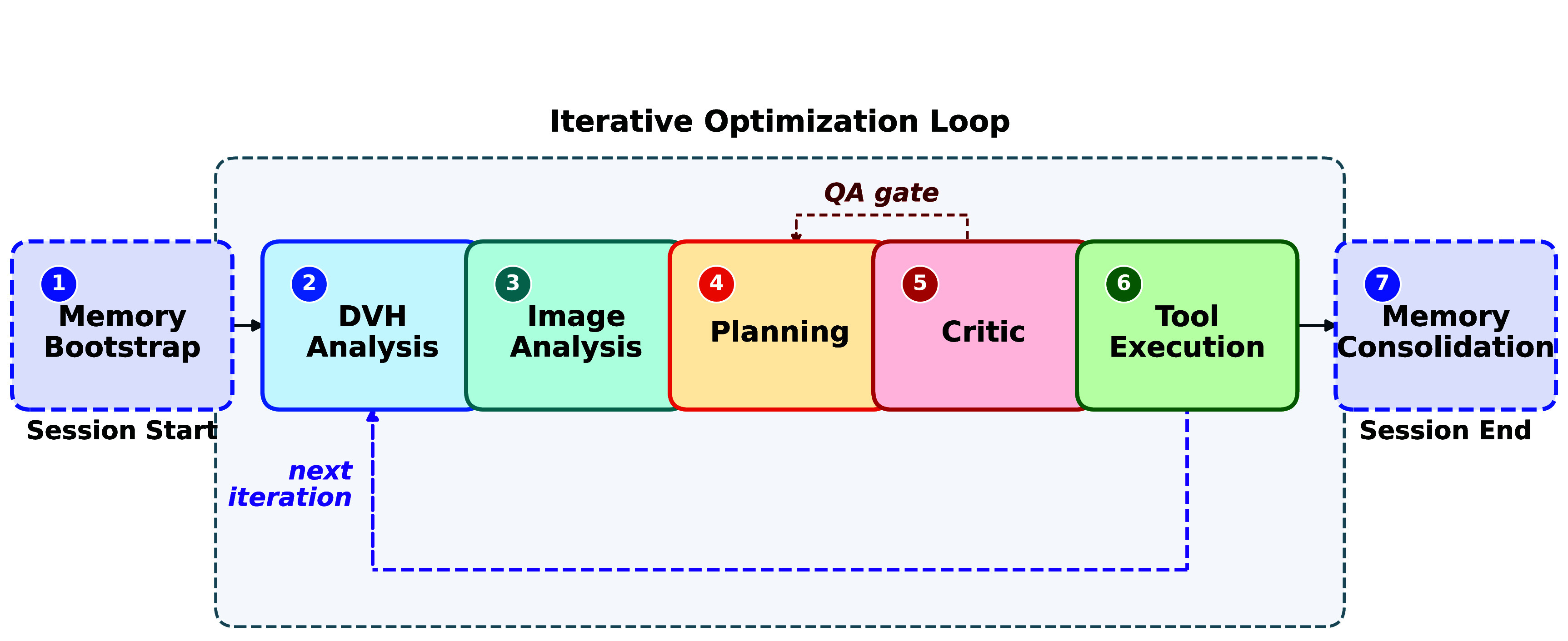
The 7-agent orchestration pipeline. Memory Bootstrap executes once per session at start. The per-iteration sequence proceeds as dose-volume histogram (DVH) Analysis $\rightarrow$ Image Analysis $\rightarrow$ Planning $\rightarrow$ Critic (quality-assurance gate) $\rightarrow$ Tool Execution, followed by treatment planning system (TPS) plan optimization and dose calculation. The Critic may reject proposed modifications, returning them to the Planning Agent for revision before proceeding to Tool Execution. Memory Consolidation executes once at session end for calibration cases only, performing knowledge extraction and write-back to the institutional knowledge base.

The platform employs an agentic orchestration layer comprising 7 specialized agents organized in a multi-agent pipeline, as illustrated in figure 3. Each agent receives a modular system prompt composed of domain knowledge sections, agent-specific instructions, and tool access specifications appropriate to its role. Agents use a HIPAA-compliant cloud-based deployment of GPT-5.2 to perform self-directed reasoning over the multi-objective optimization landscape [52, 53]. This architecture separates institutional knowledge retrieval, DVH analysis, spatial dose assessment, plan compilation, quality assurance, and tool execution into discrete reasoning stages, enabling transparent oversight of each decision point without requiring continuous human supervision.

Agent sequencing is managed by a deterministic orchestration layer that invokes each agent individually, passing agent-specific instructions and relevant upstream outputs per turn. Deterministic orchestration was chosen over an LLM-driven agentic dispatcher to maintain operational consistency across evaluation cases.

**Figure 4.**
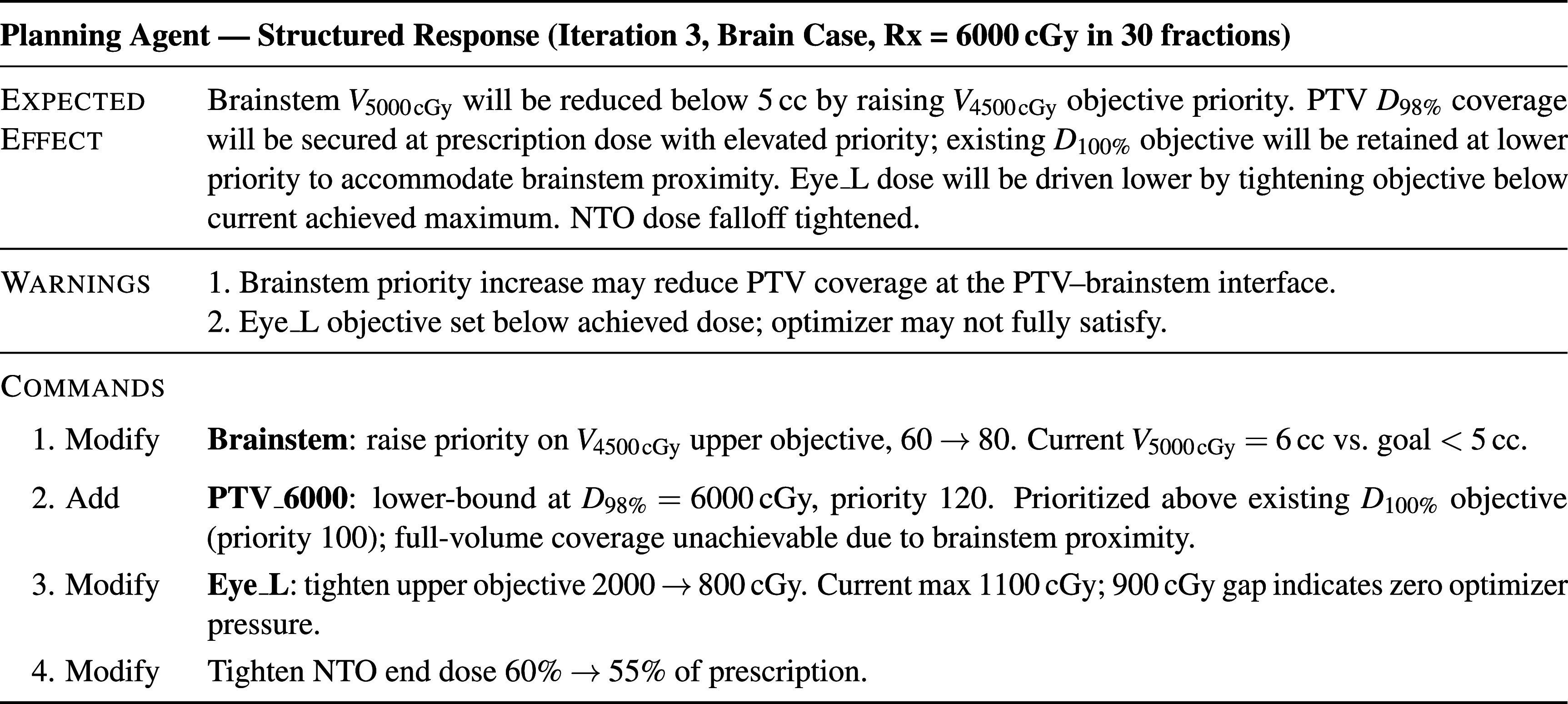
Example of a structured typed response from an agent. This particular schema required strategy articulation (Expected Effect) and risk assessment (Warnings) before executable commands, enforcing analytical reasoning prior to plan execution. The commands reference planning target volume (PTV) coverage objectives, normal tissue objective (NTO) parameters, and dose metrics such as the volume receiving at least a specified dose ($V_{x\,\mathrm{cGy}}$) and the dose to a given percentage of the structure volume ($D_{x\%}$). Rx denotes the prescription dose.

Each agent’s output is encoded as a structured typed response rather than free-form text. This format requires each agent to articulate its assessment before specifying actionable parameters, so that analytical reasoning precedes execution at every pipeline stage. Figure 4 illustrates the Planning Agent’s response schema, in which strategy articulation and risk assessment fields precede the executable command list.

The **Memory Bootstrap Agent** executes once at the start of each planning session to establish institutional context and generate initial optimization recommendations. It first retrieves the closest matching institutional experience from memory, when available, to conceptualize general planning strategies for the case. When a patient’s prescription does not exactly match stored experience, the system retrieves the closest available prior experience for that disease site based on prescription proximity. When no match is found, it falls back to embedded domain knowledge. It then retrieves clinical goals through a prioritized fallback. The system first checks for a patient-specific physician directive, then a disease-site and fractionation-specific organ-sparing directive sheet, and finally the broadest institutional safety criteria matched to the prescribed fractionation schedule. The agent is also capable of drawing on embedded read-only clinical reference material, such as Quantitative Analyses of Normal Tissue-Effects in the Clinic (QUANTEC), when formulating its recommendations [9].

The **DVH Analysis Agent** receives predicted DVH tables based on the output from the directive-conditioned dose prediction model. From the 2^nd^ iteration onward, current and prior iteration distributions are additionally provided. The agent produces per-structure dose summaries and evaluates each clinical goal against current DVH metrics, reporting regressions, critical findings, improvement opportunities, and the clinical goal achievement percentage.

The **Image Analysis Agent** receives axial CT images centered on the PTV. At the 1^st^ iteration, the agent performs anatomical analysis of structure geometry and spatial relationships. From the 2^nd^ iteration onward, dose distribution overlays are included, and the agent identifies spatial patterns including hotspots, cold spots, dose gradients, and conformality deficiencies not apparent from DVH metrics alone.

The **Planning Agent** compiles upstream analysis from the DVH Analysis and Image Analysis agents into a structured response comprising three fields (figure 4). These fields are an expected effect statement articulating the anticipated dosimetric outcome of each proposed change, a warnings section identifying potential trade-offs, and a list of executable commands with per-command rationale. The agent is instructed to identify previously unsuccessful strategies and repetitive modification patterns to guide its next approach. The platform supports optional end-user feedback, which is integrated directly into command generation when provided, although this capability was not exercised during evaluation. This agent maintains a persistent conversation history across iterations, whereas all other agent histories are re-initialized per iteration.

The **Critic Agent** functions as a quality-assurance gate that reviews the Planning Agent’s output. It evaluates proposed modification commands against a structured safety taxonomy spanning immediate rejection criteria (e.g. lower-bound objectives applied to organs at risk, removal of PTV coverage objectives), advisory warnings (e.g. priority imbalance, parameter range violations), and command consistency checks. The agent returns a 3-value verdict (approved, approved with warnings, or rejected) with per-command issue records and suggested corrections. Rejected proposals are returned to the Planning Agent with rationale for revision in a within-iteration feedback loop. Advisory warnings are carried forward into subsequent iterations to maintain quality-assurance continuity.

The **Tool Execution Agent** evaluates each modification command individually within a focused context window, verifying that the requested action is consistent with the rationale provided by the Planning Agent. It then executes validated commands as TPS scripting calls through the integration layer.

The **Memory Consolidation Agent** executes once at session end and was enabled only for the calibration cohort (disabled during evaluation to prevent test-case bias). It analyzes the full optimization trajectory, extracts generalizable insights including effective strategies, organ-sparing patterns, dose-priority relationships, and geometry-specific trade-offs, and persists these to the institutional knowledge base organized by disease site. Each insight is annotated with its subjective confidence score based on the session’s outcome. Knowledge is organized by disease site and prescription, with write access restricted to the disease-site-specific learning directory.

Agentic tool access is governed as summarized in table 1. The Tool Execution Agent holds exclusive command dispatch access, constituting the sole pathway through which plan modifications are issued to the commercial TPS.

**Table 1.** Tool access assignment for the 7-agent orchestration pipeline. Each agent is restricted to the listed access types at session initialization. Abbreviations: DVH, dose-volume histogram; TPS, treatment planning system.

Agent	Tool access	Access type
Memory Bootstrap	Plan query, memory read	Read only
DVH Analysis	None	—
Image Analysis	None	—
Planning	None	—
Critic	None	—
Tool Execution	TPS command dispatch	Dispatch only
Memory Consolidation	Memory read + write	Read + write

### 2.3. Workflow

Each planning session begins with initialization: the end-user selects a patient and identifies a clinical reference plan with appropriate beam geometry. The platform creates a new AI plan that inherits the beam arrangement and prescription parameters from the clinical reference plan. This plan starts with no prior dose distribution and no optimization objectives, ensuring that all objectives are initialized autonomously by the agentic orchestration layer. Structures are identified and classified into anatomical roles (Body, PTV, clinical target volume (CTV), gross tumor volume (GTV), OAR) using DICOM metadata and an institutional alias library. Per-OAR geometry metrics are then computed relative to the PTV, including overlap volume, nearest surface-to-surface distance, and centroid-to-centroid distance.

The Memory Bootstrap Agent uses the classified structures and prescription parameters to identify the disease site and retrieve clinical goals through the prioritized fallback described above. Then, the directive-conditioned 3D dose prediction model is invoked: the platform assembles the input tensor from TPS-extracted data, and the model predicts a dose distribution from which DVH metrics are computed per structure. Predicted DVH values serve as an achievability reference for objective initialization and modification in subsequent iterations.

Next, the iterative optimization loop refines plans toward clinical acceptance criteria and dosimetric criteria. Each iteration proceeds through DVH Analysis, Image Analysis, Planning, Critic, and Tool Execution agents in sequence. Each agent receives role-appropriate context per iteration, drawn from available sources including predicted, current, and prior DVH metrics, clinical goals, per-structure objective change summaries, and axial dose images. DVH tables presented to agents were computed at a bin interval of 100 cGy to balance dosimetric granularity with effective context window utilization.

During evaluation, clinical reference plan data was completely withheld from agent context. The platform supports optional end-user feedback through the human-in-the-loop interface, which is integrated directly into the Planning Agent’s command generation when provided. This capability was not exercised during evaluation, and all 60 cases ran fully autonomously.

Following the Tool Execution Agent’s execution, modifications are committed to the TPS through the integration layer. The platform then sequentially executes plan optimization and dose calculation, and updates with the latest 3D dose distribution, DVH metrics, achievement scores, and axial dose images. The iterative loop proceeds to the next iteration until the maximum iteration budget is reached. Upon session completion, the Memory Consolidation Agent analyzes the optimization trajectory and persists extracted insights to the institutional knowledge base (calibration cases only).

### 2.4. Directive-conditioned 3D dose prediction

The compound AI platform incorporates a directive-conditioned 3D dose prediction model as introduced in section I.6, callable by the agentic orchestration layer to supply spatially informed objective initialization during autonomous planning. The model is a hybrid 3D U-Net with a transformer bottleneck, comprising 5 encoder and 5 decoder levels with skip connections. The model was trained from scratch with randomly initialized weights on a site-agnostic cohort of 700 patients with physician directives extracted from our institutional database. The architecture and training procedure are described in our previous work [66]. As described in section I.6, the model serves as a warm-start for downstream agent tuning rather than as a final predictor, so state-of-the-art prediction accuracy is not required. Any alternative architecture or training procedure producing a dose prediction from directive-conditioned inputs would integrate identically into the compound-AI platform, so neither is a focus of the present work. The cited prior work included an optional plan-sum prediction component for multi-plan treatment courses, which we excluded here because retrospective curation of plan-sum dosimetric goals proved challenging due to inconsistent separation of plan-specific and cumulative constraints in clinical directive records.

The 7-channel input shown in table 2 conveys patient anatomy, target prescriptions, and dosimetric goals to the prediction model, corresponding to the single-plan variant of our prior work. CT images were converted from Hounsfield units (HU) to RED using our institution’s calibration curve. The 3 dosimetric goal channels were generated by spatially mapping dosimetrist goals (maximum, mean, and volumetric) to each corresponding structure’s voxels, with unspecified OAR regions assigned 95% of the plan prescription dose. The model input was built by extracting data through the TPS scripting host and resampling to 2 mm isotropic resolution. Clinical inference used a TorchScript-compiled model with sliding window inference over $160\times192\times192$ voxel patches for memory-efficient processing.

**Table 2.** Input tensor channel specification for the directive-conditioned dose prediction model. Channels 5–7 encode dosimetric goals by spatially mapping constraint values to each structure’s physical location. Unspecified OAR regions were assigned 95% of the plan prescription dose. Abbreviations: CT, computed tomography; RED, relative electron density; OAR, organ at risk; PTV, planning target volume.

Channel	Content	Encoding
1	CT image (relative electron density)	RED, normalized to $[0,\,1.0]$
2	OARs (unique random decimal per structure)	Values in $(0,\,1.0]$
3	Euclidean distance to nearest isocenter	Millimeters
4	PTVs	Gy/100
5	Dosimetric goal: maximum (spatially mapped)	Spatially mapped max dose goals
6	Dosimetric goal: mean (spatially mapped)	Spatially mapped mean dose goals
7	Dosimetric goal: volumetric (spatially mapped)	Spatially mapped dose limits at spared organ subvolumes, prioritizing voxels distant from PTV

### 2.5. Patient cohort

To evaluate this platform, we conducted a retrospective study under an institutional review board (IRB)-approved protocol at University of Texas Southwestern Medical Center . We assembled a cohort of 60 previously treated clinical plans spanning 3 anatomically distinct disease sites. Within each site, cases were evenly split between IMRT and VMAT (10 each). Prescription regimens were not restricted, encompassing both hypofractionated and conventionally fractionated schemes as summarized in table 3. PTV sizes averaged $248.5 \pm 240.9$ cc (range 14.6–1311.8 cc) across the cohort, with site-specific means of $346.9 \pm 271.2$ cc (range 42.4–1311.8 cc) for brain, $136.1 \pm 117.9$ cc (range 14.6–415.8 cc) for lung, and $262.6 \pm 262.8$ cc (range 63.6–881.8 cc) for prostate. Lung laterality was distributed as 11 left-sided, 7 right-sided, and 2 midline/central. Brain laterality was distributed as 8 left-sided, 8 right-sided, and 4 midline/central. Beam arrangement was held constant between the clinical reference plan and AI plan for each case, with VMAT cases ranging from 1 to 5 arcs and IMRT cases ranging from 5 to 9 static fields depending on the original clinical plan design. All clinical reference plans served as blinded benchmarks for autonomous plan quality evaluation.

**Table 3.** Patient cohort summary by disease site. Abbreviations: Rx, prescription; Fx, fractions; Gy/fx, dose per fraction.

Site	$n$	Rx Dose range (Gy)	Rx Fx Range	Rx Gy/fx range
Brain	20	20–60	5–33	1.80–5.00
Lung	20	45–60	3–30	1.80–18.00
Prostate	20	40–79.2	5–44	1.80–9.00

Prior to evaluation, the institutional knowledge base was populated through a two-stage calibration process using an independent cohort of 20 patients (table 4). The goal of calibration was to extend the institutional knowledge base through worked examples. The platform planned each calibration case with full knowledge of the clinical reference plan, compared its own optimization decisions against that clinical plan, and recorded its resulting observations to the knowledge base for retrieval by subsequent sessions. No parameters of the dose prediction model or the language model were updated at any point in this calibration. In the first stage, each calibration case was processed with the agentic orchestration layer permitted to reference the corresponding clinical reference plan. The Memory Consolidation Agent updated the knowledge base after each case to record site-specific optimization learnings. In the second stage, a batched retrospective pass over the calibration data was performed to distill more generalized lessons across cases. Each case was passed to the Memory Consolidation Agent with a prompt oriented toward extracting transferable institutional patterns rather than session-specific observations. This calibration phase was distinct from the evaluation cohort and served to populate the institutional knowledge base before autonomous planning was assessed. During evaluation, reference plan access and Memory Consolidation writing were both disabled to prevent knowledge accumulation from test cases biasing subsequent plans within the cohort.

**Table 4.** Calibration cohort used to populate the institutional knowledge base. Abbreviations: Rx, prescription; Fx, fractions; Gy/fx, dose per fraction.

Site	$n$	Rx Dose range (Gy)	Rx Fx Range	Rx Gy/fx range
Brain	7	40.05–60	15–30	1.90–2.67
Lung	6	50–60	3–30	1.80–18.00
Prostate	7	25–70.2	5–39	1.80–5.00

Each case was processed through the platform for 5 autonomous optimization iterations without manual intervention. For fair dosimetric comparison, the optimization model, dose calculation algorithm (Anisotropic Analytical Algorithm, AAA), and calculation grid resolution (0.15 cm for stereotactic cases, 0.25 cm otherwise) were matched between AI and clinical reference plans. After the final iteration, each AI plan was normalized so that primary PTV coverage matched the corresponding clinical reference plan’s coverage, ensuring that dosimetric comparisons reflected OAR sparing differences rather than coverage discrepancies. We benchmarked AI-generated plans against the corresponding clinical reference plans using the proportion of dosimetric criteria satisfied.

### 2.6. Evaluation metrics and baselines

We assessed plan quality using an achievement score defined as the proportion of dosimetric criteria satisfied, expressed as a percentage. Each structure-directive pair was evaluated against DVH metrics from the plan dose distribution to produce a binary achieved or not-met outcome. The achievement score was computed as shown in equation (1),

\begin{equation*} \text{Achievement Score} (\%) = \frac{\text{Goals Achieved}}{\text{Total Goals}} \times 100.\end{equation*}

Per-structure dosimetric criteria for achievement score calculation spanned five endpoint types across PTVs and OARs: maximum and minimum point dose ($D_\mathrm{max}$, $D_\mathrm{min}$), mean dose ($D_\mathrm{mean}$), partial-volume dose ($D_\mathrm{V}$, e.g. $D_\mathrm{5cc}$ or $D_{50\%}$), and volume receiving at least a given dose ($V_\mathrm{D}$, e.g. $V_{30\%\mathrm{Rx}}$). Table 5 summarizes which endpoints apply per structure. Constraint thresholds depend on prescription and fractionation: a prostate plan’s rectum sparing may target $V_{4500\,\mathrm{cGy}}$, $V_{6000\,\mathrm{cGy}}$, and $V_{7000\,\mathrm{cGy}}$ under conventional fractionation, but $V_{1800\,\mathrm{cGy}}$, $V_{2900\,\mathrm{cGy}}$, and $V_{3850\,\mathrm{cGy}}$ for a 5-fraction prescription. Threshold values vary across sites, structures, and prescriptions, and the full combination is too large to tabulate. The table therefore reports which endpoints apply rather than their various numerical thresholds. Appendix A lists explicit thresholds used for each structure, stratified by disease site and fractionation.

**Table 5.** Scope of the dose-constraint evaluation framework by disease site, structure, and DVH endpoint. A checkmark indicates that the endpoint is used at least once for that structure within the site. Thresholds depend on prescription and fractionation, so one checkmark may represent several per-structure constraints at different dose levels or volumes (e.g. multiple $V_\mathrm{D}$ constraints on the bladder). Sub-regions (e.g. rectal wall, bladder wall, lungs-PTV) are aggregated into their parent structure’s row. Abbreviations: PTV, planning target volume; $D_\mathrm{max}$, maximum point dose; $D_\mathrm{min}$, minimum point dose; $D_\mathrm{mean}$, mean dose; $D_\mathrm{V}$, partial-volume dose (absolute or fractional, e.g. $D_\mathrm{5cc}$, $D_{50\%}$); $V_\mathrm{D}$, volume receiving at least dose $D$; L/R, left/right.

Site	Structure	$\boldsymbol{D_{\max}}$	$\boldsymbol{D_{\min}}$	$\boldsymbol{D_\mathrm{mean}}$	$\boldsymbol{D_{V}\,/\,V_{D}}$
All	PTV	$\checkmark$	$\checkmark$	—	$\checkmark$

Brain	Brainstem	$\checkmark$	—	—	$\checkmark$
	Cochlea (L/R)	$\checkmark$	—	—	$\checkmark$
	Eye (L/R)	$\checkmark$	—	$\checkmark$	—
	Lens (L/R)	$\checkmark$	—	—	—
	Optic Nerves / Chiasm	$\checkmark$	—	—	$\checkmark$
	Spinal Cord	$\checkmark$	—	—	$\checkmark$

Lung	Bronchus	$\checkmark$	—	—	$\checkmark$
	Esophagus	$\checkmark$	—	—	$\checkmark$
	Great Vessels	$\checkmark$	—	—	$\checkmark$
	Heart	$\checkmark$	—	—	$\checkmark$
	Lungs	—	—	—	$\checkmark$
	Ribs	$\checkmark$	—	—	$\checkmark$
	Spinal Cord	$\checkmark$	—	—	$\checkmark$

Prostate	Bladder	$\checkmark$	—	—	$\checkmark$
	Bowel	$\checkmark$	—	—	$\checkmark$
	Cauda Equina	$\checkmark$	—	—	$\checkmark$
	Femoral Head (L/R)	—	—	$\checkmark$	$\checkmark$
	Penile Bulb	$\checkmark$	—	$\checkmark$	$\checkmark$
	Rectum	$\checkmark$	—	—	$\checkmark$
	Sigmoid	$\checkmark$	—	—	$\checkmark$
	Urethra	$\checkmark$	—	—	—

To contextualize the autonomous planning timeline, we analyzed 14 756 treatment plans generated at our institution between 2021 and 2025 . Planning time was defined as the elapsed time from physician contour approval to planner task completion in the institutional record-and-verify system.

### 2.7. Statistical analysis

Achievement score differences between clinical reference and AI plans were assessed using the Wilcoxon signed-rank test. Subgroup comparisons by disease site and delivery technique were corrected for multiple comparisons using the Benjamini–Hochberg procedure. Statistical significance was defined as adjusted $p < 0.05$, and all reported $p$-values were two-tailed. Per-structure DVH metric differences were computed as paired deltas (AI minus reference) and reported as the mean $\pm$ standard deviation.

## 3. Results

### 3.1. Execution summary

All 60 cases completed the full 5-iteration optimization pipeline without manual intervention or pipeline failure. The cohort comprised 20 brain, 20 lung, and 20 prostate cases, evenly divided between IMRT ($n = 30$) and VMAT ($n = 30$) delivery techniques (table 3). The AI agents executed $63.5 \pm 8.7$ actions per case across 5 iterations ($12.7$ per iteration).

### 3.2. Achievement scores

As shown in table 6, AI plans demonstrated statistically significant differences in achievement scores compared to clinical reference plans ($p < 0.001$) with 35 of 60 cases improved, 13 unchanged, and 12 lower. When stratified by disease site, prostate and brain cases both showed statistically significant differences (adjusted $p = 0.026$). Lung cases, which had the highest baseline reference scores among the three sites, did not reach significance (adjusted $p = 0.165$).

**Table 6.** Achievement scores (proportion of dosimetric criteria satisfied) for clinical reference plans versus AI plans (best of 5 iterations). $\Delta$ Median is the median paired difference (%). Wilcoxon signed-rank $p$-values for subgroups are reported with Benjamini–Hochberg correction; the overall comparison is reported without correction. +/ = /− denotes the number of cases in which the AI plan scored higher, equal, or lower than the reference, respectively. Bold indicates adjusted $p < 0.05$. Abbreviations: AI, artificial intelligence; IMRT, intensity-modulated radiation therapy; VMAT, volumetric modulated arc therapy; $\Delta$, difference.

Subgroup	$n$	Reference (%)	AI Plan (%)	$\Delta$ Med (%)	$p$	+ / = / −
All Plans	**60**	$\mathbf{85.2 \pm 10.8}$	$\mathbf{89.8 \pm 9.4}$	**5.1**	$\boldsymbol{< 0.001}$	**35 / 13 / 12**

Brain	20	$84.0 \pm 10.2$	$88.9 \pm 11.7$	5.3	$\mathbf{0.026}$	11 / 5 / 4
Lung	20	$88.6 \pm 8.3$	$92.0 \pm 7.3$	4.9	$0.165$	11 / 5 / 4
Prostate	20	$82.9 \pm 13.0$	$88.6 \pm 8.7$	5.5	$\mathbf{0.026}$	13 / 3 / 4

IMRT	**30**	$\mathbf{84.3 \pm 11.8}$	$\mathbf{94.1 \pm 6.7}$	**8.5**	$\boldsymbol{< 0.001}$	**25 / 5 / 0**
VMAT	30	$86.1 \pm 9.7$	$85.6 \pm 9.9$	0.0	$0.770$	10 / 8 / 12

Brain IMRT	10	$85.9 \pm 8.8$	$95.2 \pm 5.1$	—	—	—
Brain VMAT	10	$82.1 \pm 11.5$	$82.6 \pm 13.2$	—	—	—
Lung IMRT	10	$87.3 \pm 8.7$	$96.3 \pm 4.5$	—	—	—
Lung VMAT	10	$89.9 \pm 8.1$	$87.8 \pm 7.3$	—	—	—
Prostate IMRT	10	$79.6 \pm 16.1$	$90.8 \pm 8.9$	—	—	—
Prostate VMAT	10	$86.2 \pm 8.5$	$86.4 \pm 8.4$	—	—	—

**Figure 5.**
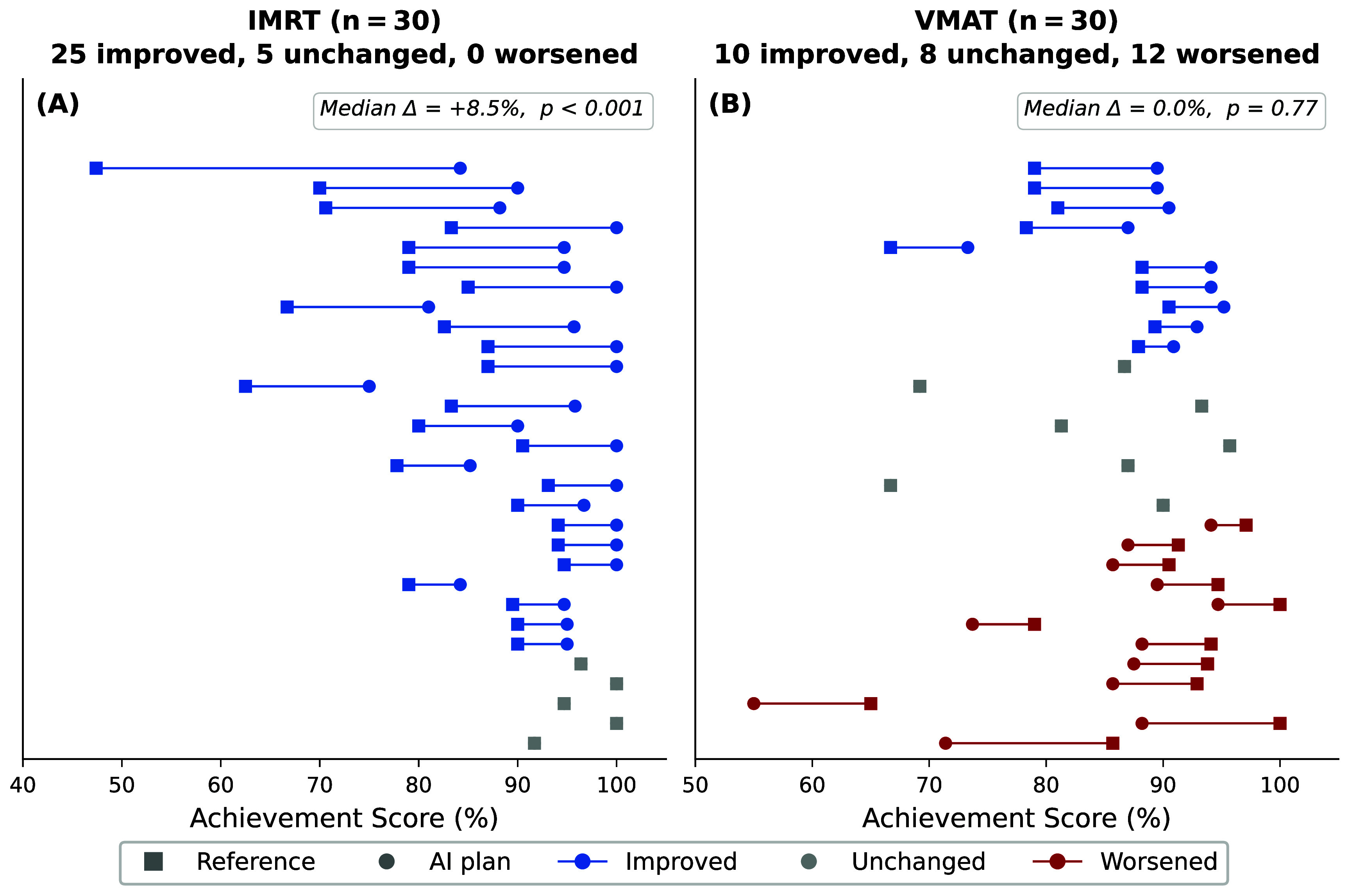
Paired achievement scores for clinical reference plans (squares) versus artificial intelligence (AI) plans (circles), stratified by delivery technique. Each line connects one patient’s reference and AI scores, colored by outcome: improvement (blue), no change (gray), or worsening (red). (A) Intensity-modulated radiation therapy (IMRT) cases showed near-universal improvement (25/30 improved, 0/30 worsened, $p < 0.001$). (B) Volumetric modulated arc therapy (VMAT) cases showed mixed results (10/30 improved, 12/30 worsened, $p = 0.770$).

The results reveal asymmetry between delivery techniques. As shown in figure 5, IMRT plans improved substantially, with 25 of 30 cases showing higher achievement scores and none worsening ($p < 0.001$). This improvement was uniform across all three disease sites. VMAT plans showed comparable performance to clinical reference plans ($p = 0.770$), with 10 of 30 improved and 12 worsened.

### 3.3. Achievement score trajectories

**Figure 6.**
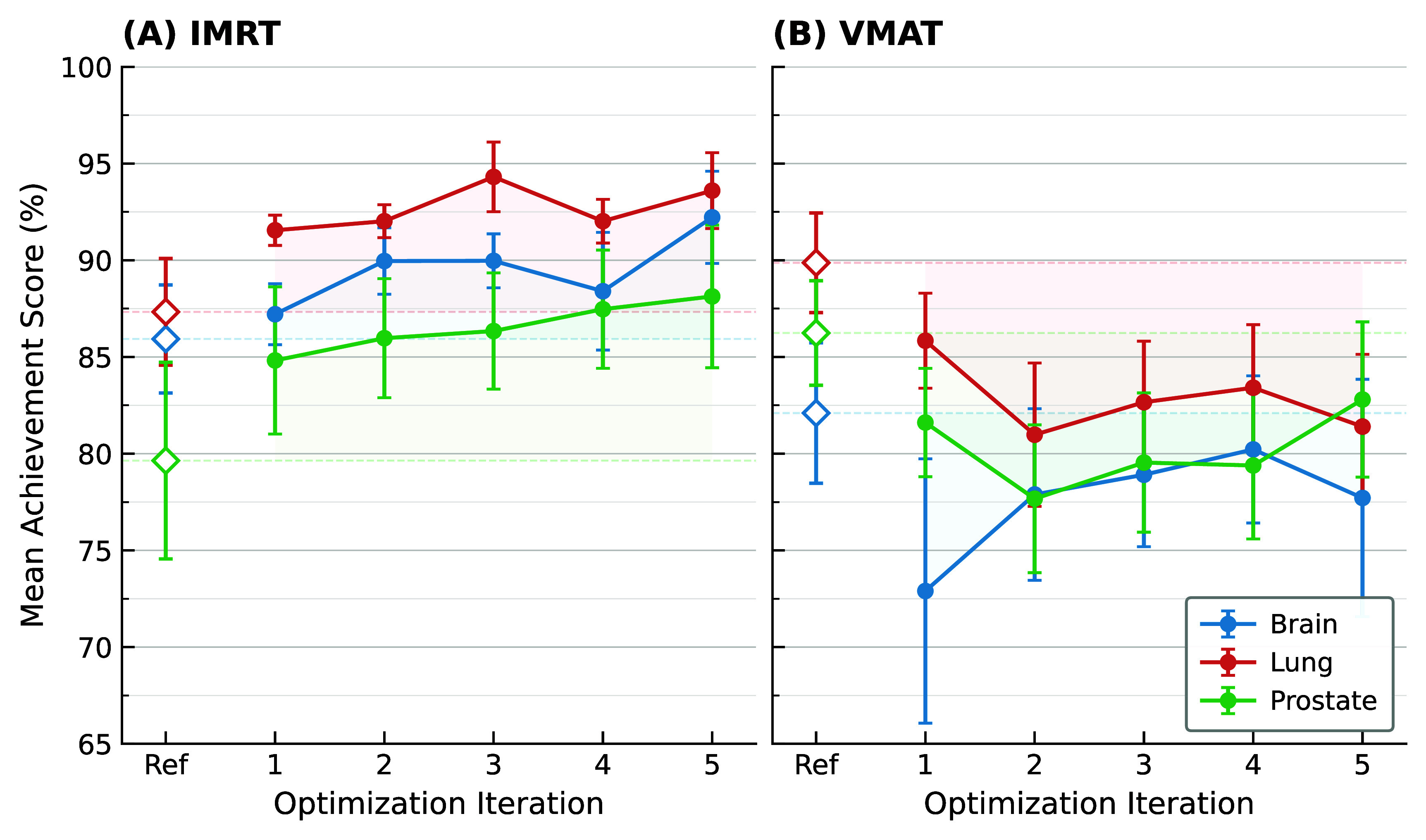
Achievement score trajectories across five optimization iterations, stratified by delivery technique. The 5-iteration budget was held fixed across disease sites for controlled evaluation. Diamond markers indicate clinical reference baselines. (A) Intensity-modulated radiation therapy (IMRT) plans improved progressively across all three disease sites, exceeding their clinical reference baselines from iteration 1 onward. (B) Volumetric modulated arc therapy (VMAT) plans showed variable iteration behavior, with achievement scores comparable to clinical reference baselines ($p = 0.770$). Shaded regions indicate deviation from the clinical reference. Error bars denote $\pm$ 1 standard error ($n = 10$ per subgroup). Across all 60 cases, the best achievement score was reached at iteration $2.47 \pm 1.44$, and 53.3% of cases peaked by iteration 2. These observations are consistent with the budget exceeding typical convergence requirements.

A fixed 5-iteration optimization budget was applied across all disease sites for controlled evaluation. IMRT plans improved progressively across all five optimization iterations at every disease site (figure 6). All three IMRT trajectories exceeded their respective clinical reference baselines from iteration 1 onward and continued to gain through iteration 5, with lung IMRT showing the largest single-iteration jump between iterations 2 and 3. Prostate IMRT improved monotonically across all five iterations.

VMAT plans showed different iteration dynamics than IMRT. VMAT achievement scores were comparable to clinical reference baselines throughout ($p = 0.770$), though the progressive improvement pattern observed for IMRT was not replicated. Lung VMAT scores were highest at iteration 1, brain VMAT peaked at iteration 4, and prostate VMAT remained stable across iterations. These technique-dependent iteration patterns suggest that VMAT optimization may benefit from different convergence strategies than the fixed five-iteration budget used in this study.

Across all 60 cases, the best achievement score was reached at iteration $2.47 \pm 1.44$ (median 2), and 53.3% had peaked by the second iteration. IMRT plans benefited more from continued iteration than VMAT plans ($2.67 \pm 1.40$ versus $2.27 \pm 1.48$). Lung cases peaked earliest at $2.10 \pm 1.25$, with 50% reaching their best score at iteration 1, whereas brain and prostate cases required additional iterations ($2.70 \pm 1.38$ and $2.60 \pm 1.67$). Lung VMAT peaked earliest of all subgroups at $1.50 \pm 0.85$, with 70% of cases peaking at iteration 1.

### 3.4. Per-structure dosimetric comparison

Target coverage was preserved in AI plans, with PTV $D_{95\%}$ differing by $-0.86$ %Rx as in table 7. OARs present in fewer than 10 cases ($n < 10$) were omitted from the table due to their limited sample size, as well as for brevity. AI plans achieved OAR dose reductions across all three disease sites. The largest improvements were observed for prostate OARs (femorals, penile bulb, bladder) and brain OARs (cochleas, eyes), while lung OAR reductions were smaller but consistent. Serial organ constraints were maintained, with spinal cord and brainstem $D_\mathrm{max}$ showing no clinically meaningful differences. Thirteen OARs achieved 100% directive satisfaction across all evaluated plans ($n \unicode{x2A7E} 3$), including spinal cord, penile bulb, bowel, femoral heads, and lungs.

**Table 7.** Per-structure DVH metric differences between AI plans (best of 5 iterations) and clinical reference plans, stratified by disease site. $\Delta = \mathrm{AI} - \mathrm{Reference}$ reported as the mean paired difference; positive values indicate higher dose or volume in AI plans, negative values indicate lower. PTV metrics reflect the primary (highest-dose) target only. Both AI and clinical reference plans were normalized to the clinical reference plan prescription coverage, so $D_{95\%}$ values are not necessarily uniformly equal to 100% of the prescription dose. Per-site sections aggregate the 20 cases per disease site; $n$ denotes the number of cases for which the structure was contoured and evaluable. OARs present in fewer than 10 cases were omitted from the table due to their limited sample size. Abbreviations: AI, artificial intelligence; OAR, organ at risk; PTV, planning target volume; SD, standard deviation; Rx, prescription; $D_\mathrm{max}$, maximum point dose; $D_\mathrm{min}$, minimum point dose; $D_\mathrm{mean}$, mean dose; $D_{95\%}$, dose to 95% of the structure volume; $D_\mathrm{5cc}$, dose to the highest-dose 5 cm$^3$; $V_\mathrm{D}$, volume receiving at least dose $D$; % vol, percent volume.

Site	Structure	Metric	${n}$	${\Delta}$	SD	Unit
All	PTV	$D_\mathrm{max}$	60	$-2.50$	$10.92$	%Rx
	PTV	$D_\mathrm{min}$	60	$+1.43$	$10.15$	%Rx
	PTV	$D_{95\%}$	60	$-0.86$	$1.82$	%Rx
	Spinal Cord	$D_\mathrm{max}$	41	$-0.37$	$5.83$	%Rx

Brain	Brainstem	$D_\mathrm{max}$	20	$-1.16$	$5.41$	%Rx
	Cochleas (L+R)	$D_\mathrm{max}$	17	$-4.27$	$10.72$	%Rx
	Cochleas (L+R)	$D_\mathrm{mean}$	17	$-4.43$	$9.26$	%Rx
	Eyes (L+R)	$D_\mathrm{max}$	19	$-3.01$	$9.90$	%Rx
	Eyes (L+R)	$D_\mathrm{mean}$	19	$-2.08$	$4.37$	%Rx
	Optics (Nerves + Chiasm)	$D_\mathrm{max}$	20	$-2.55$	$11.00$	%Rx

Lung	Esophagus	$D_\mathrm{max}$	20	$+0.67$	$7.14$	%Rx
	Esophagus	$D_\mathrm{mean}$	20	$-0.45$	$2.39$	%Rx
	Heart	$D_\mathrm{max}$	20	$-4.52$	$13.26$	%Rx
	Heart	$D_\mathrm{mean}$	20	$-0.83$	$1.36$	%Rx
	Lungs (L+R)	$D_\mathrm{mean}$	20	$-0.77$	$1.33$	%Rx
	Lungs (L+R)	$V_\mathrm{20\,Gy}$	20	$-0.42$	$1.48$	% vol

Prostate	Bladder	$D_\mathrm{max}$	20	$+1.06$	$2.74$	%Rx
	Bladder	$V_{50\%\mathrm{Rx}}$	20	$-3.39$	$9.16$	% vol
	Bowel	$D_\mathrm{max}$	16	$-2.35$	$3.31$	%Rx
	Bowel	$D_\mathrm{5cc}$	16	$-2.11$	$4.70$	%Rx
	Cauda Equina	$D_\mathrm{max}$	12	$-1.59$	$4.32$	%Rx
	Femoral Heads (L+R)	$D_\mathrm{max}$	20	$-5.34$	$7.94$	%Rx
	Femoral Heads (L+R)	$V_{30\%\mathrm{Rx}}$	20	$-1.19$	$10.68$	% vol
	Penile Bulb	$D_\mathrm{max}$	19	$-4.21$	$7.82$	%Rx
	Penile Bulb	$D_\mathrm{mean}$	19	$-3.97$	$5.65$	%Rx
	Rectum	$D_\mathrm{max}$	20	$-0.95$	$3.65$	%Rx
	Rectum	$V_{50\%\mathrm{Rx}}$	20	$-0.74$	$8.94$	% vol

Relative to clinical reference plans, AI plans showed a per-plan monitor unit (MU) change of $+8.1 \pm 32.3\%$ overall ($p = 0.210$), indicating that the observed dosimetric improvements did not come at the cost of substantially increased delivery complexity.

### 3.5. AI agent behavior analysis

Across 60 plans, the platform executed 2,859 objective modifications during the refinement phase (iterations 2–5), averaging $47.6 \pm 7.7$ per plan and $11.9 \pm 2.9$ per refinement iteration (table 8). Absolute dose perturbation magnitudes decreased from 217 cGy at iteration 2 to 130 cGy at iteration 5, a 40% reduction, while the number of modifications per iteration remained stable at approximately 12. New objective additions declined from 2.1 per plan at iteration 2 to 0.7 at iteration 5, indicating that the objective landscape was established early and subsequent iterations focused on parameter tuning. Modifications were balanced between OAR (58%) and PTV (42%) objectives overall, with early iterations favoring OAR modifications (63% at iteration 2) and later iterations approaching parity (54% OAR at iteration 4). Site-specific dynamics were consistent across disease sites (table 9). By objective type, maximum-dose (32.3%) and volumetric (31.6%) objectives were modified most often and together accounted for nearly two-thirds of all actions (table 10). Lower-bound (26.6%) and mean-dose (9.5%) objectives were modified less frequently. Maximum-dose objectives carried the largest dose perturbations ($226 \pm 389$ cGy). Lower-bound objectives carried the largest priority changes ($26.5 \pm 16.9$).

**Table 8.** Per-iteration optimization dynamics during the refinement phase (iterations 2–5) across all 60 plans. Total modifications include objective additions, parameter modifications, and removals. Dose and priority changes reflect absolute magnitudes of parameter perturbations. OAR/PTV percentages are based on all modification actions targeting identified structures. Abbreviations: Iter., iteration; Mods, modifications; Obj., objectives; $\Delta$, change; Pri., priority; OAR, organ at risk; PTV, planning target volume.

Iter.	Mods/Plan	Added	Removed	$|\Delta\mathrm{Dose}|$ (cGy)	$|\Delta\mathrm{Pri.}|$	%OAR/%PTV
2	$12.7 \pm 2.8$	2.1	0.1	$217 \pm 291$	$22.8 \pm 17.9$	63/37
3	$11.5 \pm 3.0$	1.6	0.1	$177 \pm 365$	$21.7 \pm 16.1$	58/42
4	$11.8 \pm 2.7$	1.1	0.1	$152 \pm 318$	$19.9 \pm 12.5$	54/46
5	$11.7 \pm 3.1$	0.7	0.1	$130 \pm 250$	$19.9 \pm 13.7$	58/42

2–5	$11.9 \pm 2.9$	1.4	0.1	$168 \pm 309$	$21.0 \pm 15.1$	58/42

**Table 9.** Site-specific optimization dynamics during the refinement phase (iterations 2–5). Each site comprises 20 plans (10 IMRT, 10 VMAT). Values represent per-plan per-iteration means across all refinement iterations. Abbreviations: Mods, modifications; $\Delta$, change; Pri., priority; OAR, organ at risk; PTV, planning target volume; IMRT, intensity-modulated radiation therapy; VMAT, volumetric modulated arc therapy.

Site	Mods/Plan	Added	Removed	$|\Delta\mathrm{Dose}|$ (cGy)	$|\Delta\mathrm{Pri.}|$	%OAR/%PTV
Brain	$11.2 \pm 3.4$	1.5	0.1	$156 \pm 305$	$25.6 \pm 16.6$	48/52
Lung	$12.0 \pm 2.4$	1.0	0.1	$181 \pm 331$	$20.3 \pm 14.8$	62/38
Prostate	$12.6 \pm 2.8$	1.8	0.1	$167 \pm 290$	$17.8 \pm 13.1$	64/36

**Table 10.** Optimization dynamics by objective type during the refinement phase (iterations 2–5) across all 60 plans. Lower = lower-bound objectives; Max = upper-bound at 0% volume (maximum dose constraints); Volumetric = upper-bound at $ > $0% volume; Mean = mean dose objectives. Percentages represent each type’s share of all classified actions at each iteration. Dose and priority change magnitudes reflect absolute perturbation values. Abbreviations: Iter., iteration; $\Delta$, change; Pri., priority.

Type	% of Actions					$|\Delta\mathrm{Dose}|$ (cGy)	$|\Delta\mathrm{Pri.}|$
	Iter. 2	Iter. 3	Iter. 4	Iter. 5	Average		
Lower	27.4%	26.2%	27.0%	25.6%	26.6%	$82 \pm 95$	$26.5 \pm 16.9$
Max	33.1%	32.5%	30.6%	33.0%	32.3%	$226 \pm 389$	$22.4 \pm 15.9$
Volumetric	26.8%	33.1%	34.3%	32.4%	31.6%	$177 \pm 350$	$17.0 \pm 11.5$
Mean	12.7%	8.2%	8.1%	9.0%	9.5%	$151 \pm 140$	$12.2 \pm 7.4$

All	100.0%	100.0%	100.0%	100.0%	100.0%	$168 \pm 309$	$21.0 \pm 15.1$

### 3.6. Qualitative comparison

**Figure 7.**
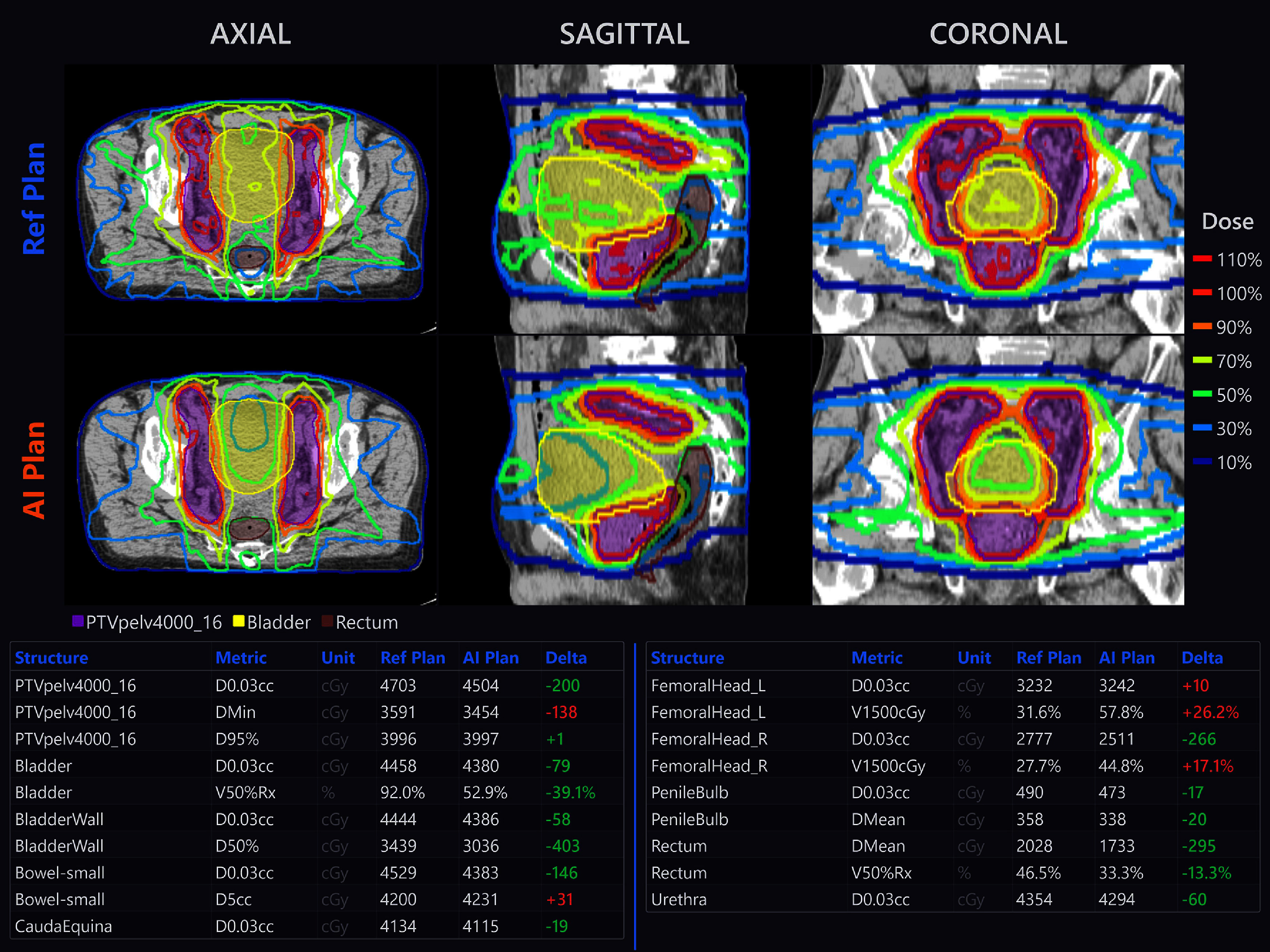
A prostate with nodes case that receives 40 Gy in 16 fractions. Top row: clinical reference plan. Bottom row: artificial intelligence (AI) plan. Dose is overlaid on axial computed tomography (CT) images with the planning target volume (PTV) (purple), bladder (yellow), and rectum (brown) contours displayed. Below the images is a dose-volume histogram (DVH) metric comparison between the two plans. Overall, the AI plan achieved reduced bladder and rectum doses without significant cost to PTV coverage.

**Figure 8.**
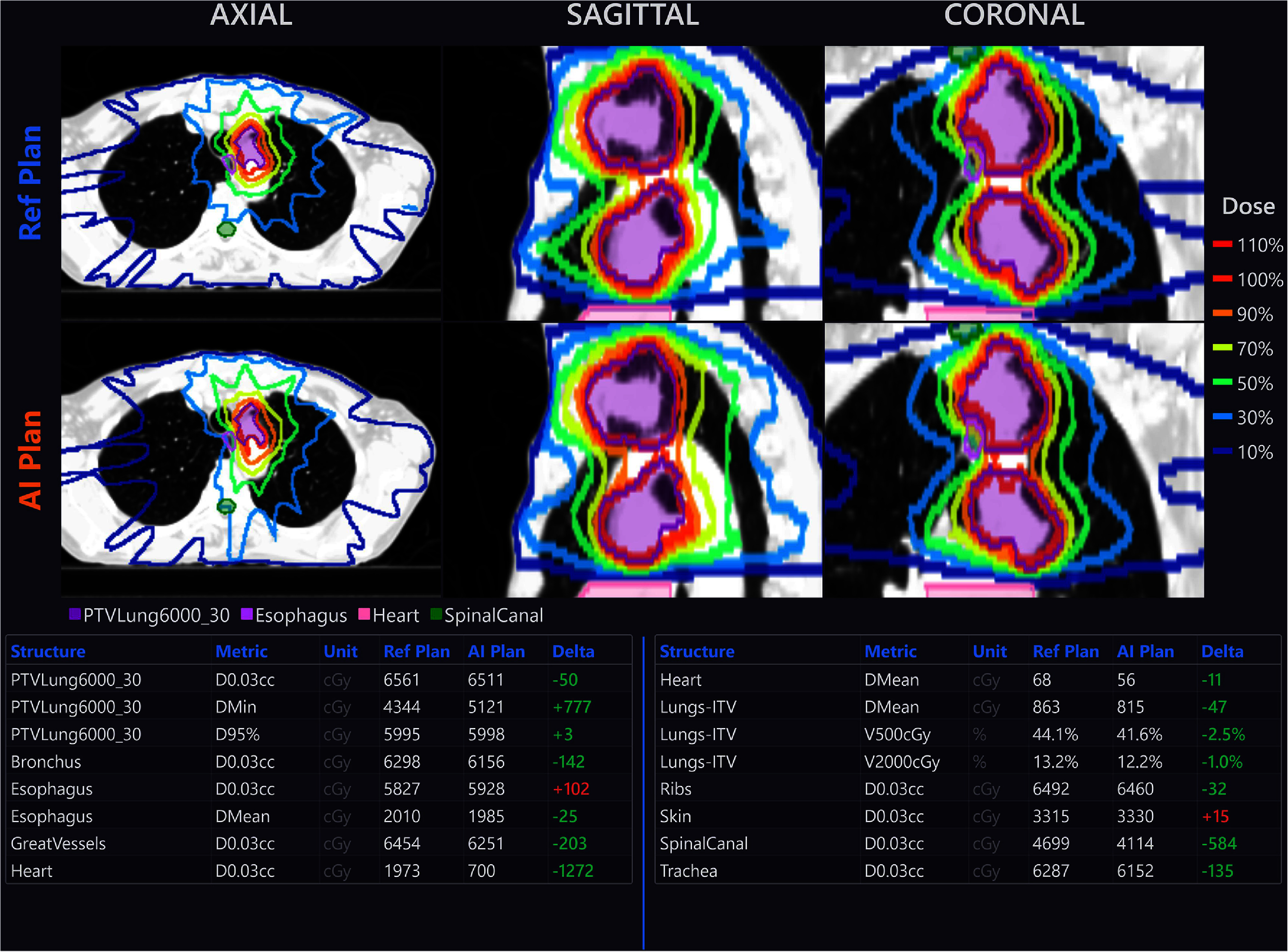
A lung case that receives 60 Gy in 30 fractions. Top row: clinical reference plan. Bottom row: artificial intelligence (AI) plan. Dose is overlaid on axial computed tomography (CT) images with the planning target volume (PTV) (purple), esophagus (light purple), heart (pink), and spinal canal (green) contours displayed. Below the images is a dose-volume histogram (DVH) metric comparison between the two plans. The AI plan outperformed the clinical reference plan in most metrics, most particularly in the minimum dose delivered to the PTV.

**Figure 9.**
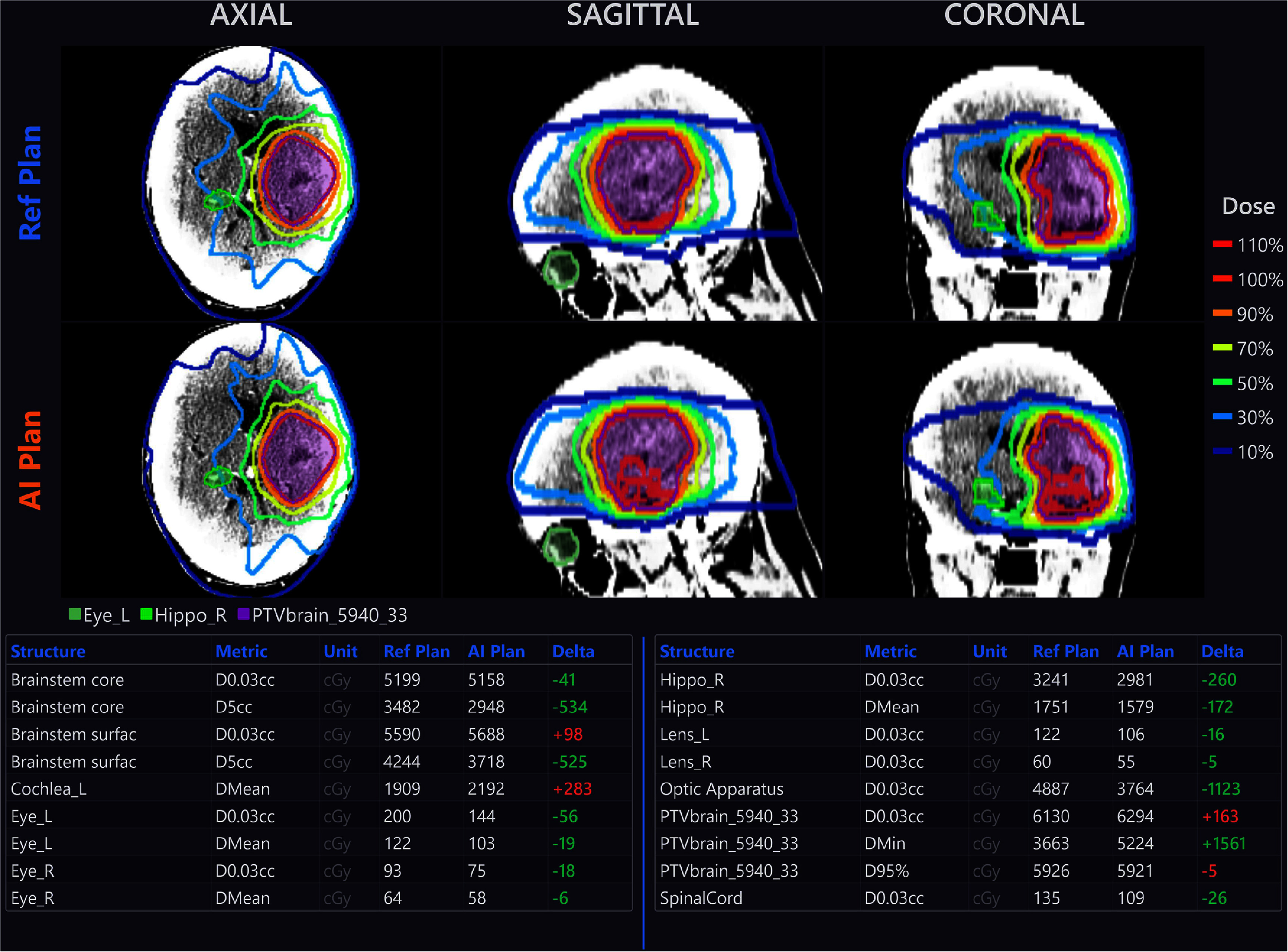
A brain case that receives 59.4 Gy in 33 fractions. Top row: clinical reference plan. Bottom row: artificial intelligence (AI) plan. Dose is overlaid on axial computed tomography (CT) images with the planning target volume (PTV) (purple), left eye (green), and right hippocampus (light green) contours displayed. Below the images is a dose-volume histogram (DVH) metric comparison between the two plans. The AI plan substantially improved the minimum dose to the PTV while also substantially reducing dose to the optics.

Figure 7 shows a prostate with nodes case receiving 40 Gy in 16 fractions. Figure 8 shows a lung case receiving 60 Gy in 30 fractions. Figure 9 shows a brain case receiving 59.4 Gy in 33 fractions. Supplementary figures for six additional cases (two per disease site with unique prescription doses) are provided in appendix B.

### 3.7. Computational performance and cost

The platform’s orchestration layer, dose prediction model, and commercial TPS integration all ran on a single TPS-compatible workstation. LLM agent reasoning was performed via API access to our institution’s cloud-based deployment, so local LLM compute was not required on the workstation. The dose prediction model ran on CPU and generally completed each prediction in under 1 min, but can be accelerated to 1–2 s with a GPU such as an NVIDIA RTX 4090. Total pipeline duration was $101.1 \pm 63.5$ min per case, of which AI agent reasoning consumed $5.2 \pm 1.7$ min per iteration and the remainder was commercial TPS optimization and dose calculation. Shared API key usage may have inflated variability in agent reasoning time. For comparison, we collected planning turnaround times for 14 756 treatment plans generated at our institution between 2021 and 2025. Across these plans, the time from physician contour approval to planner task completion was $66.5 \pm 47.7$ h with a median of 53.4 h (interquartile range: 26.3–96.8 h).

We calculated cost for the LLM’s API-based pricing at the time of evaluation. LLM inference cost was estimated as $2.16 $\pm$ $0.22 per case ($572\,143 \pm 58\,992$ tokens).

## 4. Discussion

### 4.1. Summary of key findings

AI-generated plans achieved $89.8 \pm 9.4\%$ of dosimetric criteria compared with $85.2 \pm 10.8\%$ for clinical reference plans, suggesting that the compound AI platform satisfied a greater proportion of dosimetric criteria than clinical reference plans. These results spanned brain, lung, and prostate cases with prescription doses ranging from 20 to 79.2 Gy in 3 to 44 fractions, using both VMAT and IMRT techniques. The iterative feedback loop appeared beneficial, as achievement scores improved from $84.0 \pm 12.4\%$ at iteration 1 to $86.0 \pm 13.1\%$ at iteration 5. This trajectory suggests that iterative self-correction through the agentic orchestration layer may represent a key mechanism within the compound AI platform. In addition, the platform achieved these results without site-specific handling in the software, demonstrating the feasibility of a site-agnostic approach that may match or exceed expert plan quality.

The objective dynamics data suggested that the platform engaged in substantive plan refinement rather than superficial parameter perturbation. Dose perturbation magnitudes decreased across successive iterations while the number of modifications per iteration remained stable, a pattern consistent with coarse-to-fine optimization. The shift from OAR-dominated modifications in early iterations toward a more balanced OAR/PTV distribution in later iterations suggested a two-phase strategy. The platform prioritized organ sparing first and then redirected attention toward target coverage as OAR constraints were progressively satisfied.

### 4.2. Comparison with existing approaches

The present platform differed from the 6 published LLM-based planning systems across 5 dimensions [56–61]. First, existing systems were restricted to 1 or 2 disease sites with relatively small and homogeneous cohorts. The present platform was validated across 3 anatomically distinct sites (60 cases), enabled by the directive-conditioned 3D dose prediction model and RAG-based knowledge retrieval. Second, our system coordinated 7 specialized agents with quality assurance steps in each iteration. Third, to the best of our knowledge based on the information provided in each paper, all 6 systems required optimization objectives to be initialized from non-agentic inputs (e.g. historical plans, curated templates, or manual specification) [56, 57, 59, 60]. In comparison, our platform derived patient-specific initial objectives autonomously from dose prediction. Fourth, no competing system integrated 3D dose prediction as an agentic tool. Fifth, we are the first to develop clinically deployable software that enables autonomous agentic planning with structured end-user oversight.

A key architectural distinction was the role of the directive-conditioned 3D dose prediction model. It served as a warm-start tool that generated predicted DVH values from which initial optimization objectives were autonomously derived, requiring no pre-existing objectives or manual initialization. Since the model served as a warm start rather than a final predictor, absolute dose prediction accuracy was less critical than providing a reasonable initial configuration from which agents iteratively refined.

### 4.3. Clinical implications

From a clinical perspective, the institutional planning time analysis yielded $66.5 \pm 47.7$ h (median 53.4 h) from physician contour approval to planner task completion. By comparison, the autonomous platform completed plans in $101.1 \pm 63.5$ min (${\sim}$1.7 h) across 5 iterations, representing approximately 2.5% of the institutional mean. If used to augment rather than replace the manual workflow, the agentic pipeline would add only this marginal time relative to the existing human effort. Additionally, the pipeline requires no human supervision and could be executed during off-hours to avoid any impact on the clinical schedule.

The platform may also address the inter-planner variability issue previously detailed. The institutional knowledge base accumulated optimization knowledge across sessions organized by disease site and prescription. Insights independently observed across multiple sessions received incremented confirmation through progressive confidence refinement. By encoding and consistently applying this knowledge, the platform could reduce the subjective inconsistency that underlies quality variation. Transparent agent reasoning traces may also provide junior planners with demonstrations of expert-level decision-making, which could accelerate competency development.

More broadly, the workforce shortages previously described underscore the need for planning automation. By encoding institutional knowledge through the RAG system and applying it consistently across cases, the platform could extend expert-level plan quality to settings with limited planning capacity. A site-agnostic autonomous planning platform that followed dosimetric criteria could provide these settings with consistent planning quality, and plan automation for routine cases may enable the limited expert workforce to focus on cases requiring human judgment.

### 4.4. Experimental design considerations

Several parameters were held fixed for controlled evaluation, including a uniform 5-iteration budget, beam geometry copied from the clinical reference plan, a fixed agent-calling order for reproducibility, and no use of the human-in-the-loop steering interface. In clinical deployment, these constraints could be relaxed: the iteration budget could adapt to convergence behavior, beam geometry could be selected from templated arrangements, and the platform could terminate early upon achieving clinical acceptance criteria. The efficacy of real-time expert steering on plan quality would be interesting to investigate in future works.

### 4.5. Limitations

We acknowledge several study limitations. This study’s cohort comprised a modest number of relatively standard cases, sufficient for a preliminary evaluation of the agent. Future work should extend evaluation to additional disease sites and to complex scenarios that reflect real clinical practice, such as multi-site treatments or re-irradiation. In evaluation, the achievement score captured only formally documented dosimetric criteria and may not have reflected the full scope of clinical intent. In practice, physicians may convey implicit objectives through verbal communication or informal channels, and some preferences may represent tacit clinical knowledge understood among experienced planners but not explicitly documented. Plans that satisfied all documented criteria may therefore have underperformed relative to the treating physician’s complete set of preferences. Future implementations could incorporate physician-specific preference profiles to improve adherence to individual clinical styles.

The platform’s multi-component architecture introduces potential for cascading errors across the agent pipeline. LLM agents may propose contradictory or clinically inappropriate objectives if reasoning context is insufficient, a risk inherent to any LLM-based system. The multi-agent design partially addresses this through cross-verification between specialized agents, though these safeguards do not eliminate the risk entirely.

Several additional platform-level limitations were identified. The calibration cases used to populate the institutional knowledge base were not thoroughly curated for optimal representativeness. The knowledge consolidation mechanism was disabled during evaluation to prevent test-set contamination, though in clinical practice the platform could continuously refine its institutional knowledge as each new case is planned. The platform did not include functionality for agents to create optimization-helper structures, thereby restricting agents to structures already present in the patient’s structure set. The Image Analysis Agent viewed a fixed set of axial slices rather than selecting slices based on clinical context.

The observed IMRT/VMAT performance asymmetry may also reflect a TPS API constraint specific to direct aperture optimization. VMAT optimization in the commercial TPS used in this study directly optimized multi-leaf collimator (MLC) leaf positions and dose rates at each control point through progressive stages that began with large aperture adjustments and gradually reduced the permitted magnitude of change. Once early stages committed to aperture shapes, subsequent stages could only apply small refinements. The scripting API permitted objective modifications only before the onset of each new optimization, not between internal stages, preventing agents from adjusting objectives as the optimizer progressively constrained the delivery solution. In contrast, IMRT optimized over fluence maps with uniform freedom across iterations, and MLC leaf sequencing occurred only after convergence. This asymmetry in optimizer accessibility may partly explain why VMAT plans did not benefit from agentic refinement to the same degree as IMRT plans.

### 4.6. Future directions

Several extensions are architecturally feasible. Careful curation of calibration patients across disease sites could yield a stronger and more generalizable knowledge base. Agentic creation of and optimization with optimization-helper structures represents a natural extension that could be implemented as an agentic tool. Additionally, allowing the Image Analysis Agent to select which axial slices to evaluate could provide more targeted spatial feedback during plan refinement. Lastly, the modular multi-agent architecture imposes no inherent constraints on the number, specialization, or interaction patterns of agents, allowing the platform to be extended or reconfigured as clinical requirements and AI capabilities evolve.

## 5. Conclusion

We developed a compound AI platform for end-to-end autonomous radiotherapy treatment planning that integrated multi-agent LLM orchestration, directive-conditioned 3D dose prediction, RAG-based institutional knowledge retrieval, and real-time TPS integration. Integrating dose prediction as an agent-invoked tool enabled autonomous objective initialization, eliminating the dependence on curated templates and manual specification that constrained prior LLM-based planning systems. Seven specialized agents navigated the multi-objective optimization landscape through structured clinical reasoning across five fully autonomous iterations per case. Evaluated on 60 retrospective cases across 3 disease sites with heterogeneous prescriptions (20–79.2 Gy in 3 to 44 fractions), the platform achieved 89.8$\pm$9.4% of dosimetric criteria versus 85.2$\pm$10.8% for clinical reference plans ($p < 0.001$), with pronounced improvement for IMRT plans (94.1$\pm$6.7% vs 84.3$\pm$11.8%, $p < 0.001$) and comparable VMAT performance (85.6$\pm$9.9% vs 86.1$\pm$9.7%, $p = 0.770$). The site-agnostic design required no site-specific reconfiguration, and each plan iteration in the autonomous pipeline completed in $20.2\pm12.7$ min, of which agentic reasoning contributed $5.2\pm1.7$ min. These results established the feasibility of fully autonomous, universal RT treatment planning through compound AI. The modular multi-agent architecture imposes no inherent constraints on agent specialization or platform scope, allowing the system to be extended as clinical requirements and AI capabilities evolve.

## Data Availability

The data cannot be made publicly available upon publication because they contain sensitive personal information. The data that support the findings of this study are available upon reasonable request from the authors. General dose constraints used for plan evaluation available at https://doi.org/10.1088/3049-477X/ae7978/data1.
